# Delayed Onset Urticaria in Depressive Patients with Bupropion Prescription: A Nationwide Population-Based Study

**DOI:** 10.1371/journal.pone.0080064

**Published:** 2013-11-14

**Authors:** Li-Yu Hu, Chia-Jen Liu, Ti Lu, Tsung-Ming Hu, Chia-Fen Tsai, Yu-Wen Hu, Cheng-Che Shen, Yu-Sheng Chang, Mu-Hong Chen, Chung-Jen Teng, Huey-Ling Chiang, Chiu-Mei Yeh, Vincent Yi-Fong Su, Wei-Shu Wang, Pan-Ming Chen, Tzeng-Ji Chen, Tung-Ping Su

**Affiliations:** 1 Department of Psychiatry, Kaohsiung Veterans General Veterans Hospital, Kaohsiung, Taiwan; 2 Department of Psychiatry, Yuli Veterans Hospital, Yuli, Taiwan; 3 Division of Hematology and Oncology, Department of Medicine, Taipei Veterans General Hospital, Taipei, Taiwan; 4 Institute of Public Health, National Yang-Ming University, Taipei, Taiwan; 5 Department of Internal Medicine, National Yang-Ming University Hospital, Yilan, Taiwan; 6 Department of Psychiatry, Taipei Veterans General Hospital, Taipei, Taiwan; 7 Cancer Center, Taipei Veterans General Hospital, Taipei, Taiwan; 8 Department of Psychiatry, Chiayi Branch, Taichung Veterans General Hospital, Chiayi, Taiwan; 9 Division of Allergy, Immunology, and Rheumatology, Department of Internal Medicine, Shuang Ho Hospital, New Taipei City, Taiwan; 10 Taipei Medical University, New Taipei City, Taiwan; 11 Division of Oncology and Hematology, Department of Medicine, Far Eastern Memorial Hospital, New Taipei City, Taiwan; 12 Department of Psychiatry, Far Eastern Memorial Hospital, New Taipei City, Taiwan; 13 Department of Psychiatry, National Taiwan University Hospital, Taipei, Taiwan; 14 Department of Family Medicine, Taipei Veterans General Hospital, Taipei, Taiwan; 15 Department of Chest Medicine, Taipei Veterans General Hospital, Taipei, Taiwan; 16 Department of Psychiatry, Su-Ao and Yuanshan Branch, Taipei Veterans General Hospital, Taipei, Taiwan; 17 National Yang-Ming University, Taipei, Taiwan; University of British Columbia, Canada

## Abstract

**Background:**

Bupropion, which is widely used in patients with depressive disorder, may cause allergic reactions. However, the real prevalence of these side effects may be overlooked and underreported due to the delayed onset phenomenon.

**Objective:**

This study aimed to estimate the real incidence of bupropion-induced urticaria and clarify the delayed onset phenomenon.

**Methods:**

We conducted a nationwide cohort study between 2000 and 2009 using Taiwan’s National Health Insurance Dataset. Among 65,988 patients with depressive disorders, we identified new users of bupropion with depressive disorders (bupropion cohort, n = 2,839) and matched them at a ratio of 1:4 regarding age and sex (non-bupropion matched cohort, n = 11,356). The risk of urticaria was compared between the two cohorts.

**Results:**

The risk of urticaria occurrence was higher in bupropion users than in matched controls within 4 weeks of starting the medication (risk ratio 1.81; 95% confidence interval 1.28–2.54; *p* = 0.001). The occurrence of urticaria in the bupropion cohort were more frequent on Days 15–28 than Day 1–14 (*p* = 0.002). Cox proportional hazards model showed that a history of urticaria was an independent risk factor for developing bupropion-induced urticaria.

**Conclusions:**

Of the antidepressants, bupropion may pose a higher risk of drug-induced urticaria, and this condition might be ignored due to the delayed onset phenomenon. Depressive patients with a history of urticaria are at higher risk of the adverse drug reaction. This study emphasizes the need for increased clinical awareness of this adverse outcome to bupropion use.

## Introduction

Bupropion hydrochloride is a popular psychotropic drug that inhibits norepinephrine and dopamine reuptake with minimal effect on serotonin. It is prescribed to millions of patients worldwide for the treatment of major depressive disorders and bipolar depression. In 1997, bupropion was approved by the U.S. Food and Drug Administration (FDA) for use as a smoking cessation aid[1,2].

The U.S. prescribing information for bupropion discloses serious allergic reactions as potential side effects, including urticaria, angioedema, erythema multiforme, Stevens-Johnson syndrome, and even anaphylactic shock. However, these allergic reactions have been rarely reported with bupropion[3]. Two large-scale studies were designed to examine the safety of bupropion as a treatment for smoking cessation in England and France, respectively[4,5]; however, assessment of allergic reactions was not emphasized in these studies. In addition, adverse effects of bupropion in depressive patients, which had been highly associated with urticaria[6], is rarely mentioned in the literature.

To date, only case reports have described bupropion-induced allergic reactions, and it is important to note that clinical features of delayed onset urticaria seems to be present in most of these patients[7-10]. Some studies have postulated that delayed onset urticaria may be overlooked and underreported[9,11] because patients who take bupropion may receive treatment for bupropion-induced delayed onset urticaria by other physicians or hospitals[9,11].

To address these inadequacies in the literature and to assess the pattern of bupropion-induced urticaria, we designed a nationwide population-based study to investigate the actual incidence and timing of new onset urticaria in patients with depression who receive bupropion prescriptions. 

## Patients and Methods

### Data Sources

 The Taiwan National Health Insurance (NHI) program offers a comprehensive, unified, and universal health insurance program to all citizens. The NHI program covers more than 96% of the country’s population and has contracted with 99% of all hospitals and clinics in Taiwan[12]. The NHI dataset covers comprehensive medical care, including complete data on outpatient visits, hospitalizations, diagnostic codes, examinations, and prescriptions. Multiple NHI databases (e.g., NHI enrolment files, claims data, and prescription drug registry) are managed and publicly released by the National Health Research Institute, Taiwan. The Bureau of National Health Insurance and the National Health Research Institute regulations guarantee patient confidentiality, and data that may have contained identifiable information were encrypted. Our study received full review by our local institutional review board (Veterans General Hospital Institutional Review Board NO. : 2013-01-035BC) and our institutional review board has waived the need for written informed consent from the participants. 

### Patient Population

 We conducted a retrospective cohort study from January 1, 2000 to December 31, 2009. The presence of depressive disorders was defined as any defined in the International Classification of Diseases, 9th revision, and the clinical modification (ICD-9-CM) codes 296.2X-296.3X, 300.4, and 311.X[13,14]. We also collected information on all types of antidepressants which were available in Taiwan. Antidepressants were classified according to the World Health Organisation (WHO) Anatomical Therapeutic Chemical (ATC) classification. 

For the study cohort, we identiﬁed 65,988 patients with depressive disorders who had received a first prescription for antidepressants (ATC code N06A) available in Taiwan between January 1, 2000 and December 31, 2009. Patients with a new prescription of bupropion were assigned to the bupropion cohort. Patients who had received bupropion before 2000 or under 20 years of age were excluded. We used the date on which the bupropion treatment was ﬁrst prescribed as the index date. Within the same observational period, for each of the 2,839 depressive patients taking bupropion, 4 insured people in the comparison cohort with other antidepressants use matched for age, sex, comorbidities, and index date were selected. The same exclusion criteria were applied to the matched comparison cohort. The other antidepressants used in the comparison cohort included fluoxetine, paroxetine, sertraline, citalopram, escitalopram, fluvoxamine maleate, venlafaxine, duloxetine, milnacipran, mirtazapine, trazodone, moclobemide, imipramine, amitriptyline, doxepin, maprotiline, clomipramine, dothiepin. The comparison group included 11,356 patients. In our study, the follow-up began on the index date and will end on 4 weeks later. 

We defined patients with an urticaria occurrence as those under ICD-9-CM codes 708.x within 4 weeks of receiving a bupropion prescription. Patients with recent urticaria within one month before receiving the new prescription of bupropion were excluded from the study. A history of urticaria was defined as urticaria occurring at least one month before the index date. The medical history could be traced because the NHIRD in Taiwan was established in 1996.

Patient comorbidities at baseline were identiﬁed. Comorbidities included autoimmune diseases (ICD-9-CM code 279.x), liver diseases (ICD9-CM codes 570-573), diabetes mellitus (ICD-9-CM codes 250), chronic kidney diseases (ICD-9-CM codes 580-593), and HIV/AIDS (ICD-9-CM code 042).

Each study patient was tracked until one of the following conditions was met: a diagnosis of urticaria (ICD-9-CM codes 708.x), follow-up was censored at the time of loss to follow-up, death, the patient withdrew from the NHI, or the follow-up period elapsed (4 weeks after index date).

In addition, to clarify whether other antidepressants could have the same delayed onset phenomenon on urticaria occurrence, we compared urticaria occurrence in patients receiving a new bupropion prescription with patients receiving new prescriptions for all other antidepressants available in Taiwan within the next four weeks as a further analysis. 

### Statistical Analyses

Urticaria occurrence was considered as the primary outcome variable in this study and was calculated for two different time periods after the depressive patients receiving the new antidepressants prescription, i.e., Day 1–14 and Day 15–28. We first compared the distribution of demographic characteristics between the bupropion cohort and the comparison group using the Mann-Whitney U test for median age and using χ^2^ test for sex and baseline comorbidities. The cumulative incidence of urticaria for the two cohorts were calculated by the Kaplan-Meier method, with the log-rank test being used to examine differences between cohorts. Cox proportional hazards model was used to identify risk factors for urticaria occurrence in depressive patients with bupropion prescription within four weeks. We implemented in both the univariate and multivariable fashion to identify risk factors for the urticaria occurrence after bupropion use. Variables included in the model were sex, age, comorbidities, and history of urticaria. The qualifying criterion for inclusion in the multivariate analysis was a result in the univariate-analysis with a *P* value of less than 0.1. In constructing Cox models, the follow-up began on the index date. The study patients who withdrew (including those who died) from the NHI program were censored. If the patient did not leave the NHI program and encountered no urticaria occurrence, the date of censoring was the date of the end of follow-up. We validated the Cox regression model by checking whether the assumption of proportionality held. The results of Scaled Schoenfeld Reiduals test shown the proportionality of hazards assumption was met in this analysis (Table S1). Finally, in order to make the diagnosis of urticaria more specific, the subgroup of urticaria patients diagnosed by dermatologic specialists were determined.

The Perl programming language (version 5.12.2) extracted and computed data. Microsoft SQL Server 2005 (Microsoft Corp., Redmond, WA, USA) was used for data linkage, processing, and control sampling. IBM SPSS (version 19.0 for Windows; IBM Corp., New York, NY, USA) and SAS statistical software (version 9.2; SAS Institute Inc., Cary, NC, USA) were used for all statistical analyses. Results of comparisons with a *p* value less than .05 were considered statistically significant.

## Results

### Study Population Characteristics

 Characteristics of patients in the bupropion and comparison cohort are shown in Table 1. Of the study population, 2,839 of 65,988 (4.3%) patients with depressive disorders had received bupropion and 11,356 of 65,988 (17.2%) patients were selected to match the bupropion cohorts based on age and sex from January 1, 2000 to December 31, 2009. The median age of the subjects was 41 years (interquartile range: 31–52 years). The majority of patients in both cohorts were female (60.16%). Liver diseases and diabetes mellitus, found in 33.3% and 20.3% of patients, respectively, were the two most common observed comorbidities. A past history of urticaria was recorded in 31.32% of patients. There were no baseline statistical differences in comorbidities and history of urticaria between the groups.

**Table 1:** Characteristics of depressive patients with a bupropion prescription and the matched cohort.

Characteristics	Bupropion cohort (*n* = 2,839)		Matched cohort (*n* = 11,356)	*p* value
	Total no. (%)		Total no. (%)	
Median age (interquartile range)	41(31–54)		41(31–54)	
20–39	1,276(44.95)		5,104(44.95)	1.000
40–59	1,050(36.98)		4,200(36.98)	
≥ 60	513(18.07)		2,052(18.07)	
Sex				
Male	1,131(39.84)		4,524(39.84)	1.000
Female	1,708(60.16)		6,832(60.16)	
Comorbidities				
Autoimmune diseases	313 (11.03)		1145 (10.08)	0.139
Liver diseases	958(33.74)		3,724(32.79)	0.335
Diabetes mellitus	591(20.82)		2,242(19.74)	0.200
Chronic kidney diseases	323(11.38)		1,368(12.05)	0.325
AIDS	4(0.14)		31(0.27)	0.204
History of urticaria	899(31.67)		3,517(30.97)	0.474

AIDS: acquired immunodeficiency syndrome.

### Incidence of Urticaria

**Figure 1:**
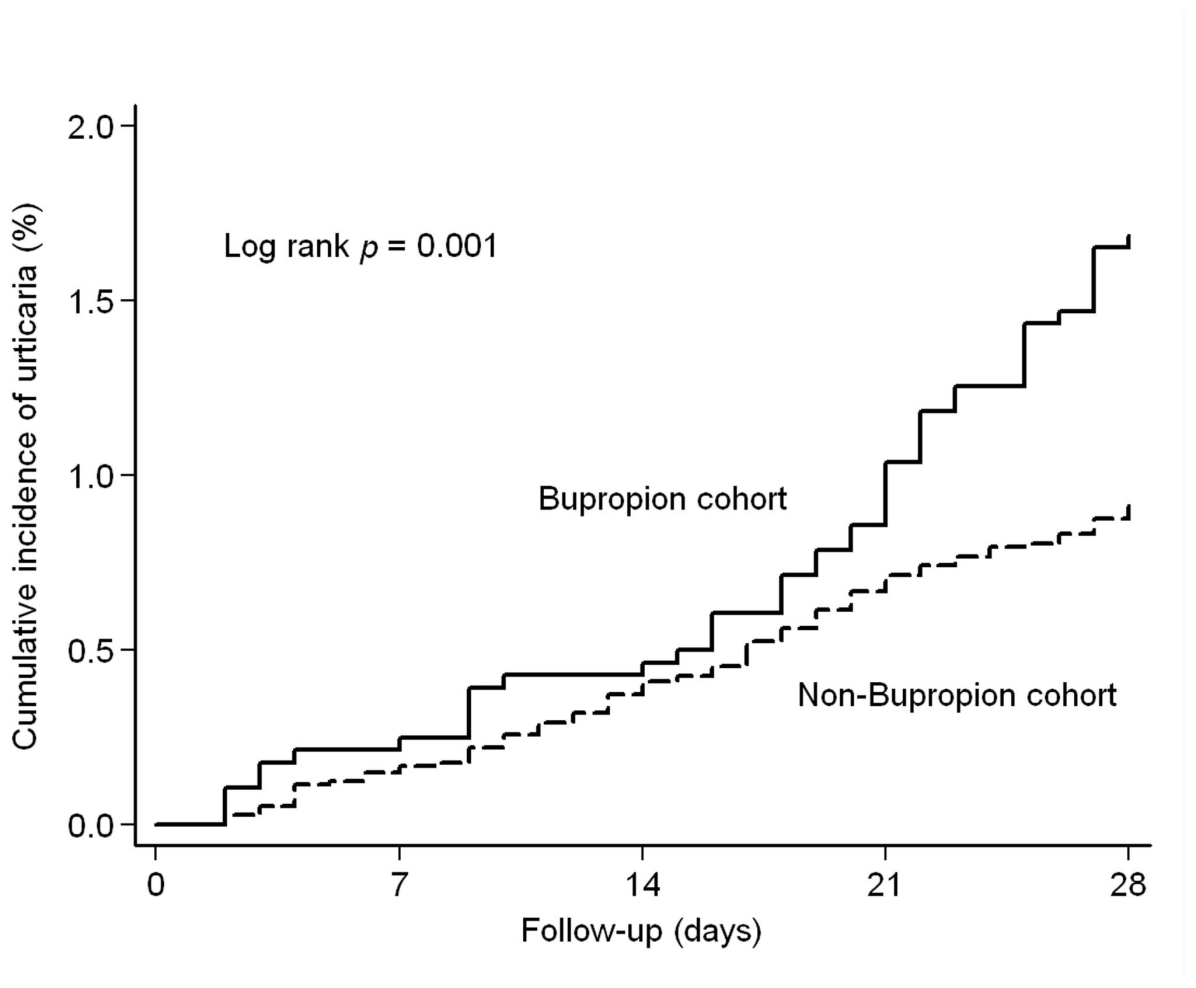
The cumulative incidences of urticaria in depressive patients with and without bupropion prescription. Follow-up(days) =0 indicates the initiation of antidepressants use.

Figure 1 shows the cumulative incidences of urticaria occurrence across all participants. The urticaria risk within 4 weeks was significantly higher for patients in the bupropion cohort (cumulative incidence, 16.56‰) than for patients in the comparison cohort (9.16‰) (risk ratio, 1.81; 95% CI 1.28–2.54, *p* = 0.001) (Table 2). We also stratified patients by age and gender and found that bupropion use was associated with higher urticaria risk in patients under 40 years of age (risk ratio, 2.25; 95% CI 1.41–3.60, *p* < 0.001), but not in patients over 40 years of age. The use of bupropion was associated with a higher risk of urticaria occurrence in both males and females. 

**Table 2:** Incidence of urticaria occurrence in depressive patients within the first 4 weeks of taking the antidepressants.

	Bupropion cohort	Matched cohort	Risk ratio	*p* value
	*n* (‰)	*n* (‰)	(95% CI)	
Total	47(16.56)	104(9.16)	1.81 (1.28–2.54)	0.001
Age				
20–39	27(21.16)	48(9.40)	2.25(1.41–3.60)	< 0.001
40–59	15(14.29)	39(9.29)	1.54(0.85–2.78)	0.151
≥ 60	5(9.75)	17(8.28)	1.18(0.44–3.17)	0.748
Sex				
Male	16(14.15)	33(7.29)	1.94 (1.07–3.51)	0.026
Female	31(18.15)	71(10.39)	1.75 (1.15–2.65)	0.008

CI: confidence interval

### Early and Delayed Onset Urticaria

 As shown in Table 3, we compared the cumulative incidences of urticaria occurrence in the Day 1–14 and the Day 15–28 between bupropion and matched cohorts. The results indicated that delayed onset urticaria occurred more frequently for patients in the bupropion cohort (cumulative incidence, 11.98‰) than for patients in the non-bupropion cohort (5.11‰) (risk ratio, 2.34; 95% CI 1.54–3.57, *p* < 0.001). 

**Table 3:** Comparisons of early and delayed onset urticaria occurrence.

	Bupropion cohort	Matched cohort	Risk ratio	*p* value
	*n* (‰)	*n* (‰)	(95% CI)	
Total	47(16.56)	104(9.16)	1.81 (1.28–2.54)	0.001
Early onset (Day 1–14)	13(4.58)	46(4.05)	1.13(0.61-2.09)	0.696
Delayed onset (Day 15–28)	34(11.98)	58(5.11)	2.34(1.54-3.57)	<0.001

### Risks Factors for Urticaria

 We next performed univariate and multivariable analyses to predict urticaria development in the bupropion cohort (Table 4). We found that a history of urticaria (HR 3.03, 95% CI 1.7–5.4, *p* < 0.001) was the only independent risk factor for urticaria occurrence following bupropion use.

**Table 4:** Analyses of risk factors for urticaria in depressive patients after bupropion use.

Variables	Univariate analysis			Multivariable analysis		
	HR	95% CI	*p* value	HR	95% CI	*p* value
Age	0.98	0.96–1.00	0.101	0.99	0.97–1.01	0.367
Male sex	0.78	0.43–1.43	0.424			
Comorbidities						
Autoimmune diseases	0.75	0.27–2.09	0.580			
Liver diseases	0.74	0.39–1.40	0.358			
Diabetes mellitus	0.35	0.13–0.98	0.046	0.38	0.13–1.10	0.075
Chronic kidney disease	1.59	0.74–3.40	0.232			
AIDS	0.05	0.00–2.12x10^13^	0.861			
History of urticaria	2.94	1.65–5.24	< 0.001	3.02	1.69–5.39	<0.001

HR: hazard ratio; CI: confidence interval; AIDS: acquired immunodeficiency syndrome.

### Delayed onset Urticaria in depressive patients with all types of newly antidepressants prescription

 To clarify whether other antidepressants could have the same delayed onset phenomenon on urticaria occurrence, we compared the cumulative incidences of urticaria in depressive patients with patients receiving all other types of new antidepressant prescriptions in Taiwan (Table 5). Bupropion-associated urticaria occurred more frequently on Day 15–28 (11.98‰) than on Day 1–14 (4.58‰) (risk ratio, 2.62, 95% CI 1.38–4.95, *p* = 0.002). Among all antidepressants, the delayed-onset phenomenon was only observed in bupropion users.

**Table 5:** The risk of urticaria occurrence among all antidepressant classes.

Antidepressant classes	Total new prescriptions	Urticaria occurrence on Day 1-14 n (‰)	Urticaria occurrence on Day 15-28 n (‰)	Risk ratio (95% CI)	*p*-value
Norepinephrine-Dopamine Reuptake Inhibitors					
Bupropion	2,839	13(4.58)	34(11.98)	2.62 (1.38–4.95)	0.002
Serotonin-Norepinephrine Reuptake Inhibitors					
Venlafaxine	7,240	24(3.31)	35(4.83)	1.46 (0.87–2.45)	0.151
Duloxetine	1,752	7(4.00)	5(2.85)	0.71 (0.23–2.25)	0.563
Milnacipran	658	5(7.60)	2(3.04)	0.4 (0.08–2.05)	0.452
Selective Serotonin Reuptake Inhibitors					
Fluoxetine	13,207	54(4.09)	61(4.62)	1.13 (0.78–1.63)	0.513
Paroxetine	8,454	34(4.02)	32(3.79)	0.94 (0.58–1.52)	0.805
Sertraline	10,747	39(3.63)	33(3.07)	0.85 (0.53–1.34)	0.479
Citalopram	5,973	23(3.85)	21(3.52)	0.91 (0.51–1.65)	0.763
Escitalopram	3,817	13(3.41)	13(3.41)	1 (0.46–2.15)	1.000
Fluvoxamine Maleate	3,057	10(3.27)	12(3.93)	1.2 (0.52–2.77)	0.669
Tricyclics					
Imipramine	8,296	46(5.54)	34(4.10)	0.74 (0.48–1.15)	0.179
Amitriptyline	3,426	20(5.84)	11(3.21)		0.105
Doxepin	4,018	33(8.21)	14(3.48)	0.42 (0.23–0.79)	0.005
Maprotiline	919	6(6.53)	1(1.09)	0.17 (0.02–1.38)	0.124
Clomipramine	667	4(6.00)	3(4.50)	0.75 (0.17–3.34)	1.000
Dothiepin	286	1(3.50)	2(6.99)	2 (0.18–21.93)	1.000
Monoamine Oxidase Inhibitors					
Moclobemide	3653	11(3.01)	13(3.56)	1.18 (0.53–2.63)	0.683
Others					
Mirtazapine	5164	15(2.90)	14(2.71)	0.93 (0.45–1.93)	0.853
Trazodone	14961	66(4.41)	44(2.94)	0.67 (0.46–0.98)	0.036

CI: confidence interval

### Urticaria patients among both cohorts diagnosed by dermatologic specialists

In this subgroup of dermatologist-diagnosed urtiacria among both cohorts, the higher risk for overall urticaria occurrence was similar to the results of the urtiacria patients diagnosed by general practitioners in the bupropion cohort. The bupropion use was still associated with higher urticaria risk in patients under 40 years of age (risk ratio, 2.95; 95% CI 1.48–5.86, *p* = 0.001), but not in patients over 40 years of age. The higher risk of urticaria occurrence in the bupropion cohort was significant in males but not in females (risk ratio, 1.47; 95% CI 0.74–2.92, *p* = 0.273). In addition, the delayed onset trend of urticaria occurrence was still found in this subanalysis (risk ratio, 2.73; 95% CI 1.42–5.25, *p* = 0.002). Detailed results are shown in the Tables S2-S3. 

## Discussion

To the best of our knowledge, the present study is possibly the largest study to analyze urticaria risk in patients with depression after starting a bupropion prescription. There are several important findings from the nationwide study. First, the unexpected high risk for urticaria occurrence was observed in patients with depression who received bupropion, especially in patients under 40 years of age. Second, the risk of urticaria occurrence in bupropion cohort were significantly higher in Day 15–28 than Day 1–14 and the delayed onset trend was not observed in other antidepressants users in our study. Finally, a history of urticaria may be an important predictor of urticaria development after taking bupropion. The findings may arouse public interest regarding safety issues in bupropion use. 

In order to examine the safety of bupropion use, two large-scale studies were previously conducted to quantify the incidence of events reported for patients who were prescribed bupropion and to identify any previously unrecognized adverse drug reactions in England and France[4,5]. In the study conducted in England, data were derived from questionnaires sent to general practitioners at least 6 months following the first prescription. However, the study achieved a response rate of only 48.1% in the patients prescribed bupropion, and therefore a recall and selection bias could not be avoided. In the French study, the safety profile of bupropion use was monitored through a spontaneous report system; thus, the results may be underestimated. The design of those previous studies may explain why the incidence of bupropion-induced urticaria was much lower than that found in our study. Although those studies provide insight into the association between bupropion administered for smoking cessation and urticaria, the association between bupropion administered for depression and unitcaria is rarely mentioned in the literature, and importantly depression has been shown to be strongly associated with urticaria[6]. In addition, our study relied on a claim-based dataset, and therefore the data related to the medical charge, such as antidepressant use, were precise. A diagnosis of urticaria would not be omitted even if the patients had received treatment at different hospitals or times. 

Several smaller reports have shown that nearly all of the antidepressants seemed to be associated with an onset of urticaria, including fluoxetine[15], paroxetine[16], sertraline[17], escitalopram[17], venlafaxine[18], bupropion[5,7,9,10,19-25], mirtazapine[18], and tricyclic antidepressants[26]. However, very few attempts have been made to compare the occurrence of urticaria among the various antidepressants. In our study, the results showed that bupropion use was associated with the highest risk of urticaria development and the delayed onset phenomenon of urticaria occurrence was only shown in the bupropion users compared with all other antidepressant prescriptions in Taiwan assessed.

In addition to bupropion-induced urticaria[21-23], many smaller studies have described a relationship between bupropion use and allergic reactions, including angioedema[20], erythema multiform[24], Stevens-Johnson syndrome[27], and serum sickness-like reactions[7-11,25]. Moreover, most of the studies noticed delayed onset characteristics of these allergic reactions. Although some researchers had presumed that the bupropion-induced urticaria may be underestimated[9,11], unanswered reasons still remain. According to our results, we hypothesized that the reason of underestimation may be related to the delayed onset phenomenon of bupropion-induced urticaria. In addition, bupropion-induced urticaria seldom develops alone and is sometimes accompanied with other more serious allergic reactions, including angioedema[19,20], arthralgia[8], serum sickness-like reaction[7,9-11], or symptoms of anaphylaxis, which may indicate a medical emergency. Therefore, if medical personnel do not recognize the adverse drug reaction, then patients could be put at risk for more severe drug hazards. 

The mechanisms of urticaria are thought to be associated with histamine and other mediators being released from mast cells and basophils. The higher occurrence of bupropion-induced urticaria in younger patients could be explained by immunosenescence, which is an age-related decline in immune functions. It has been reported that mast cell development declines through the aging process[28], and the number of dermal mast cells decreases with age in human subjects[29]. However, the mechanism of bupropion-induced urticaria is currently unknown. We hypothesize that the adverse drug reaction may be linked to the structure of bupropion, which is chemically similar to amfepramone. Amfepramone is considered a selective norepinephrine releasing agent, and norepinephrine may play an important role in adrenergic urticaria, which is considered to be a form of neurogenic reaction that is mainly triggered by stress. Moreover, in recent years, bupropion has been suggested to have effects as an anti-inflammatory agent by down-regulating tumor necrosis factor synthesis, which may slow the course of some inflammatory processes[30,31]. Therefore, we hypothesized that the anti-inflammation effect may delay the allergic reaction and lead to delayed onset urticaria. 

While urticaria may be confused with a variety of other dermatologic diseases that are similar in appearance and are also pruritic, it is possible that other urticaria-like skin reactions may have been misclassified under the code based on claims data. In order to make the diagnosis more specific, subgroup of the urtiacria diagnosed by dermatologic specialists in our study group were further analyzed and the higher risk for overall urticaria occurrence and delayed onset trend were similar to the results of the urtiacria patients diagnosed by general practitioners in the bupropion cohort.

Several limitations inherent in using claims databases also need to be taken into consideration. First, because patients’ identifications were blocked for protection of privacy, we have no way to assess for actual intake of prescribed antidepressants. However, such a limitation usually leads toward an under-estimation of risk. Besides, although no evidence has shown that the difference of drug compliance between bupropion and other antidepressants, it is reasonable to assume that the higher risk of delayed onset urticaria in the bupropion cohort may be associated with the influence of drug compliance. Second, drugs which may cause were not adjusted for. Although urticaria is really often caused by the many drugs including antibiotics and anticonvulsants use in our clinical experience. However, these drugs were not selected for adjustment in this study because the antibiotics and anticonvulsants were less common co-medications used by our study subjects (patients with depressive disorders). Besides, the frequency of antibiotics and anticonvulsants co-prescriptions with antidepressants is relatively low during a short follow-up period (4 weeks) in our study. Third, a number of other potential confounding factors that might affect urticaria risk were not available in the reimbursement data used in this study, such as psychosocial stresses, foods, family history of urticaria, recent travel history, exposure to environmental stimulants and smoking[32]. {Stockli, 2007 #51}Among these non-observable confounders, for the reason that the use of bupropion may be helpful to depressive patients who smoke[33], therefore, smoking may be an important non-observable confounder in our study. Finally, further investigations are needed to explore such associations in different populations and ethnic groups.

In conclusion, our study has found that patients with depressive disorders who received bupropion were at an unusually higher risk for delayed onset urticaria. Based upon our data, we suggest that more attention should be focused on the bupropion-induced urticaria in patients with depressive disorders and urge caution over the delayed onset phenomenon, especially in patients under 40 years of age and with past history of urticaria, in order to avoid more severe drug allergies as a result of prescribing the same drugs. Although more research is needed, this article serves to broaden physicians’ knowledge base when prescribing bupropion. 

## Supporting Information

Table S1 **Scaled Schoenfeld residuals test of proportional hazards.**
(DOC)

Table S2 **Incidence of dermatologist-diagnosed urticaria occurrence in depressive patients within first 4 weeks.**
(DOC)

Table S3 **Comparisons of early and delayed onset dermatologist-diagnosed urticaria occurrence.**
(DOC)
