# Supplementary material for: Delayed Onset Urticaria in Depressive Patients with Bupropion Prescription: A Nationwide Population-Based Study
Source: PLoS One. 2013 Nov 14;8(11):e80064. doi: 10.1371/journal.pone.0080064 (PMC3828225; doi:10.1371/journal.pone.0080064)
Supplement: Table S1 — Scaled Schoenfeld residuals test of proportional hazards. (DOC) [file pone.0080064.s001.doc]

**Table S1. Scaled Schoenfeld residuals test of proportional hazards.**

|  | rho | chisq | *p* value |
| --- | --- | --- | --- |
| **Univariate analysis** |  |  |  |
| Age | -0.093 | 0.298 | 0.585 |
| Male sex | 0.150 | 1.050 | 0.305 |
| Autoimmune diseases | 0.038 | 0.067 | 0.796 |
| Liver diseases | -0.102 | 0.487 | 0.485 |
| Diabetes mellitus | -0.267 | 3.350 | 0.067 |
| Chronic kidney disease | -0.276 | 3.590 | 0.058 |
| History of urticarial | -0.167 | 1.320 | 0.251 |
| **Multivariable analysis** |  |  |  |
| Age | -0.012 | 0.005 | 0.943 |
| Diabetes mellitus | -0.255 | 2.934 | 0.087 |
| History of urticarial | -0.158 | 1.160 | 0.281 |
| GLOBAL | NA | 4.543 | 0.208 |
