# Supplementary material for: Delayed Onset Urticaria in Depressive Patients with Bupropion Prescription: A Nationwide Population-Based Study
Source: PLoS One. 2013 Nov 14;8(11):e80064. doi: 10.1371/journal.pone.0080064 (PMC3828225; doi:10.1371/journal.pone.0080064)
Supplement: Table S2 — Incidence of dermatologist-diagnosed urticaria occurrence in depressive patients within first 4 weeks. (DOC) [file pone.0080064.s002.doc]

**Table S2**. **Incidence of dermatologist-diagnosed urticaria occurrence in depressive patients within first 4 weeks.**

|  | Bupropion cohort, *n*(‰) | Matched cohort, *n*(‰) | Risk ratio (95% CI) | *p* value |
| --- | --- | --- | --- | --- |
| Total | 20 (0.70) | 40 (0.35) | 2.00(1.17–3.42) | 0.010 |
| Age |  |  |  |  |
| 20–39 | 14(1.10) | 19(0.37) | 2.95(1.48–5.86) | 0.001 |
| 40–59 | 5(0.48) | 18(0.43) | 1.11(0.41–2.99) | 0.835 |
| ≥60 | 1(0.19) | 3(0.15) | 1.33(0.14–12.79) | 0.803 |
| Sex |  |  |  |  |
| Male | 9(0.80) | 10(0.22) | 3.60(1.47–8.84) | 0.003 |
| Female | 11(0.64) | 30(0.44) | 1.47(0.74–2.92) | 0.273 |

CI indicates confidence interval
