# Supplementary material for: Delayed Onset Urticaria in Depressive Patients with Bupropion Prescription: A Nationwide Population-Based Study
Source: PLoS One. 2013 Nov 14;8(11):e80064. doi: 10.1371/journal.pone.0080064 (PMC3828225; doi:10.1371/journal.pone.0080064)
Supplement: Table S3 — Comparisons of early and delayed onset dermatologist-diagnosed urticaria occurrence. (DOC) [file pone.0080064.s003.doc]

**Table S3**. **Comparisons of early and delayed onset dermatologist-diagnosed urticaria occurrence.**

|  | Bupropion cohort, *n*(‰) | Matched cohort, *n*(‰) | Risk ratio (95% CI) | *p* value |
| --- | --- | --- | --- | --- |
| Total | 20 (0.70) | 40 (0.35) | 2.00(1.17–3.42) | 0.010 |
| Early onset  (0-2 weeks) | 5(0.18) | 18(0.16) | 1.11(0.41–2.99) | 0.835 |
| Delayed onset (2-4 weeks) | 15(0.53) | 22(0.19) | 2.73(1.42–5.25) | 0.002 |
